# Data modeling as a main source of discrepancies in single and multiple marker association methods

**DOI:** 10.1186/1753-6561-3-s1-s9

**Published:** 2009-02-23

**Authors:** Mônica Corrêa Ledur, Nicolas Navarro, Miguel Pérez-Enciso

**Affiliations:** 1 Embrapa Suínos e Aves, BR 153, Km 110, 89700-000, Concórdia, SC, Brazil; 2 Dept. Ciencia Animal i dels Aliments, Facultat de Veterinaria, Universitat Autonoma de Barcelona, 08193, Bellaterra, Spain; 3 Institut Català de Recerca i Estudis Avançats (ICREA), Pg. Lluis Companys 23, 08010 Barcelona, Spain

## Abstract

**Background:**

Genome-wide association studies have successfully identified several loci underlying complex diseases in humans. The development of high density SNP maps in domestic animal species should allow the detection of QTLs for economically important traits through association studies with much higher accuracy than traditional linkage analysis. Here we report the association analysis of the dataset simulated for the XII QTL-MAS meeting (Uppsala). We used two strategies, single marker association and haplotype-based association (Blossoc) that were applied to i) the raw data, and ii) the data corrected for infinitesimal, sex and generation effects.

**Results:**

Both methods performed similarly in detecting the most strongly associated SNPs, about ten loci in total. The most significant ones were located in chromosomes 1, 4 and 5. Overall, the largest differences were found between corrected and raw data, rather than between single and multiple marker analysis. The use of raw data increased greatly the number of significant loci, but possibly also the rate of false positives. Bootstrap model aggregation removed most of discrepancies between adjusted and raw data when SMA was employed.

**Conclusion:**

Model choice should be carefully considered in genome-wide association studies.

## Background

Genome-wide association studies (GWAS) have successfully identified loci underlying several complex diseases [1,2] and quantitative traits, like height in humans [3]. The development of high density SNP maps in domestic animal species should allow the detection of QTLs for economic important traits through association studies with much higher accuracy than traditional linkage analysis. The simplest method to analyze GWAS is single marker association (SMA). Multiple marker analyses are also used to reduce the numbers of false positives and to increase power [4]. Nevertheless, the advantage of haplotype-based methods upon SMA has not yet been proven. To address this issue, we compare SMA with an haplotype-based method – Blossoc [5] – recently developed to take advantage of high density SNP maps genotyped in large sample sizes, still being fast and accurate in detecting causal loci. Simulation studies have demonstrated that this method outperforms SMA in a more complex situation such as mutation heterogeneity and complex haplotype structures [5]. We applied these two strategies to the raw data and to the data corrected for infinitesimal (polygenic), sex and generation effects to evaluate to what extent population structure (i.e., pedigree relationships) and environmental effects could affect the results.

## Methods

A total of 4665 animals with genotypes and phenotypes, the first 4 generations of the data set provided by the QTL-MAS Workshop [6], were included in the analyses.

### Raw versus corrected data

Initially, association analyses were performed using the raw data (Y = μ + SNP + e) fitted for each SNP. Next, the data were analyzed with a mixed model including the infinitesimal (a), sex (S) and generation (G) effects. Residuals from this mixed model were then used as input data in the haplotype-based analysis. For the corrected SMA, we used the same mixed model except that the SNP effect was estimated simultaneously: Y = μ + S + G + a + SNP + e. Mixed model analyses were carried out with QxPak, which employs a maximum likelihood approach [7].

### Single Marker Association (SMA)

An additive model was initially tested at each SNP. We first established a list of SNPs showing an association with p < 10^-8 ^(F-test), with the restriction that minimum distance between selected SNPs was 5 cM. When two significant SNPs were found within a 5 cM region, the most significant one was retained. However, not all of these putative QTLs are necessarily genuine QTLs. Recently, bootstrap model aggregation (bagging; [8]) was proposed to control for false positives in genome-wide analysis using complex crosses [9,10]. Hence, from the list of selected SNPs, we bootstrapped the data and we built multiple additive QTL models by forward selection. A SNP was included in the model if its p-value, conditional on all other SNPs already in the model, was lower than 10^-3^, otherwise, the model building was stopped. We ran 1000 iterates. The frequencies of each SNP in the models correspond to their support of being a true QTL, and are called bootstrap posterior probabilities (BPP; [9]). To speed-up the analysis, we considered a threshold of BPP ≥ 0.25 for assigning a true association. It is important to note that this threshold depends on the population and the phenotype and requires specific calibration by simulations. Therefore, 0.25 is here arbitrary and is likely to not properly control false positives. These results are presented in Additional file 1. SMA and bagging were run using a home made R script.

For the corrected-data SMA, an additive model was used with the same p-value threshold and minimum distance to select associated SNPs as with SMA on raw data. Significance tests in QxPak are based on likelihood ratio test. Intensive computational procedures to further control for false positives were not feasible with this mixed model approach, nor with the haplotype-based analyses.

### Multiple Marker Association (Blossoc)

Blossoc, a linkage disequilibrium (LD) association mapping tool, was used for the haplotype-based analyses. This method attempts to build 'perfect' phylogenetic trees for each marker and scores these according to non-random clustering of affected individuals, judging high-scoring areas as likely candidates for containing disease affecting variation [5]. Although initially designed for case-control studies, this method can also be applied to quantitative traits. Blossoc was designed to handle very dense sets of markers with high LD, so blocks of compatibility include several markers. We used a window of a minimum of 10 markers for building the phylogeny around markers. Blossoc generates scores for each marker, but gives a smooth curve, because neighboring markers are included to score a locus, so scores for close markers are more dependent than SMA. However, we expect that high clustering scores from Blossoc are highly correlated to small P-values from SMA, as demonstrated by Mailund et al. [5]. The Hannan and Quinn criteria (HQ), which is similar to the Bayesian Information Criterion (BIC), was used to indicate significant association [5]. Threshold was established based on corrected data and set at scores ≥15, which was reached by 7.3% of the SNPs. Selected peaks were also the local maximum in 10 cM regions along the genome.

### Comparison among approaches

The agreement between methods and between data corrections was based on the percentage of coincident associated SNPs. A coincident associated SNP pair or match was defined as a pair of associated SNPs in two analyses whose distance was shorter than 5 cM. The percentage of SNP matches was calculated as the ratio between the number of matches and the sum of the matches and the number of SNPs uniquely detected by either of the two analyses to be compared. The degree of concordance of matching SNPs was the absolute difference in cM of the estimated location of coincident SNPs between analyses.

### Computational details

Analyses were performed on a Linux server with dual Xeon processors and 8 Gb RAM. The total CPU time required for the analyses is shown in the Additional file 2.

## Results and discussion

**Figure 1:**
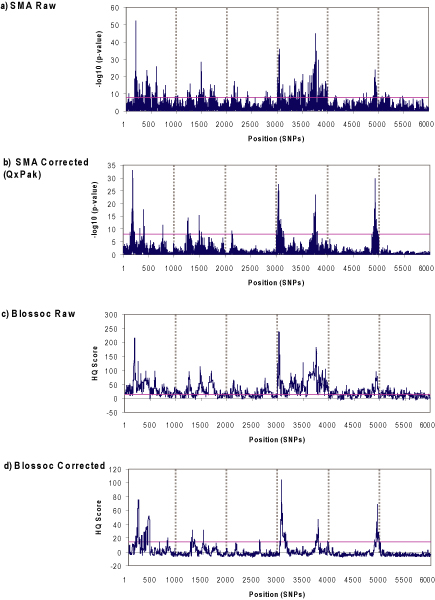
**Genome-wide association profile with single and haplotype-based association methods using different data modeling**. SMA with raw (a) and corrected data (b), and haplotype-based analysis with raw (c) and corrected data (d). The horizontal lines are the thresholds: P < 10^-8 ^for SMA and HQ score >15 for Blossoc. The vertical dashed lines separate chromosomes.

In general, both SMA and Blossoc performed similarly in identifying the most strongly associated SNPs (Figure 1), independently of the approach. About 10 loci with high additive effects were identified to affect the trait (Table 1), which had a polygenic heritability of 0.39. The most strongly associated loci were located in Chromosomes 1, 4 and 5.

**Table 1:** SNPs identified as associated with the phenotypic trait by different methods and approaches.

	**SNP (P-value)**		**SNP (HQ-score)**		**SMA Additive effect (SE)^3^**	
**Chromosome**	**SMA raw^1^**	**SMA corrected (QxPak)**	**Blossoc raw^1^**	**Blossoc corrected**	**raw**	**corrected**

1	196 (52.2)^2^	196 (33.0)	200 (215.7)	200 (75.4)	0.74 (0.05)	0.71 (0.06)
	323 (16.9)*	331 (10.4)	-	-	0.40 (0.05)	-0.69 (0.10)
	415 (23.5)	402 (17.6)	416 (98.1)	402 (52.2)	0.46 (0.05)	-0.78 (0.09)
	778 (14.9)	778 (11.5)	778 (51.7)*	778 (20.2)	0.40 (0.05)	0.40 (0.06)
2	1271 (15.8)	1270 (14.4)	1268 (94.6)	1267 (31.1)	0.36 (0.04)	0.43 (0.05)
	1483 (28.3)	1483 (15.4)	1483 (113.2)	1487 (31.2)	-0.50 (0.04)	-0.45 (0.05)
3	2149 (17.2)	2133 (9.3)	2134 (65.8)	-	-0.39 (0.04)	0.35 (0.06)
	-	-	2598 (37.9)*	2601 (17.6)	-	-
4	3048 (35.9)	3033 (27.4)	3032 (237.4)	3032 (104.4)	0.54 (0.04)	0.59 (0.05)
	3765 (44.9)	3765 (23.4)	3765 (183)	3765 (47.5)	0.62 (0.04)	0.55 (0.05)
	3953 (17.0)	-	3952 (103.2)	3952 (15.6)	0.37 (0.04)	-
5	4935 (23.7)	4935 (29.8)	4940 (94.8)	4935 (68.7)	-0.47 (0.05)	-0.63 (0.05)
**Total # SNPs**	**33/15**	**10**	**49/19**	**10**		

1 – For analysis with raw data, only SNPs that coincide with those obtained with corrected data are listed in this table. A complete list of SNPs detected using raw data is in the Additional file 1.

2 – Between brackets are the significance level for methods: -log_10 _(p-value) for SMA and HQ score for Blossoc. Threshold for SMA is P < 10^-8 ^and for Blossoc is HQ Score ≥ 15. The * shows SNPs that would not be selected using a bootstrap posterior probabilities (BPP) < 0.25 for SMA raw and an adjusted threshold for Blossoc raw <65, with a total of 15 and 19 selected SNPs for each method, respectively (Additional file 1).

3 – The direction of the additive effects is from genotype 11 to 22.

### Raw versus corrected data

Incorporating known population structure into the phenotype modeling reduced the noisiness of the association profile (Figure 1). Peaks were sharper with corrected than with raw data for both SMA and Blossoc methods. Nevertheless, raw and corrected profiles were quite correlated (r = 0.70 for SMA and r = 0.66 for Blossoc).

Three to five-fold more SNPs were selected using raw data than with corrected data (Table 1). Nonetheless, all SNPs selected with corrected data were recovered in the analyses with raw data within each method. Disagreement, therefore, is mostly due to these additional SNPs. Selection seems therefore more liberal on raw data. Additional procedures to control for false positives are clearly required. The bootstrap model aggregating (bagging) is expected to control such false positives. When applied to SMA on raw data, the bagging reduced considerably the list of putative QTLs from 33 to 15 (with a BPP ≥ 0.25). All but two of these had BPPs higher than 0.6. Those two were not recovered in the corrected data, nor were four SNPs with medium to high BPPs, but the other SNPs were also selected using the mixed model approach (Additional file 1). For Blossoc, an adjusted threshold for raw data (>65) reduced the associated SNPs from 49 to 19.

### Comparison between methods

Only ~50% of the SNPs detected using raw data coincide between SMA and Blossoc methods. This percentage increased to 67% when using corrected data. The disagreement between the corrected methods was due to 4 SNPs: SNP 331 was detected with SMA (QxPak). This SNP showed high LD with SNP 402 (D' ≈ 1, r^2 ^= 0.3), which was highly associated with the phenotype. The equivalent SNP in the SMA raw (323) showed low BPP (0.1). Therefore, SNP 331 is probably a false positive. SNP 3952 was detected with Blossoc and also with SMA raw (BPP = 0.99), suggesting that it is a true QTL. The SNP identified in the beginning of Chromosome 3 (2133) by the SMA method (BPP = 0.89) was also detected with Blossoc on raw data, but was not selected with Blossoc on corrected data. This SNP showed a well defined peak with corrected Blossoc, close to the significance threshold (HQ = 13). Therefore, SNP 2133 is probably also a true QTL. These results indicate that the thresholds defined here for both SMA and Blossoc on corrected data might be too conservative. Another SNP towards the middle end of this chromosome (2601) was detected only with Blossoc. This was the most distinct result found between methods (Figure 1) and could reflect a difference between single and haplotype-based methods, although the possibility of it being a false positive can not be discarded.

The agreement in matching SNPs was lower when using raw data (maximum distance of 4.1 cM), and increased greatly when corrected data was used (maximum distance of 0.4 cM). Nevertheless, the establishment of a threshold is a complicated issue and has a profound influence on the results, especially when methods with different nature of scores are compared. Our results indicate that 8 SNPs are confidently associated to the trait, as found by all methods evaluated here with a small variation in cM among coincident SNPs.

### Interactions within and between loci and with sex

We also fit more complex models incorporating dominance and epistasis (see Additional file 3 for methods). Dominance was not significant in any SNP. Most significant epistatic pairs are in Table 2. SNP 3295 showed a suggestive effect (P-value = 2 × 10^-5^) of interaction with sex. The additive effects of males and females were similar in magnitude but of opposite sign.

**Table 2:** Top significant epistatic interactions with P < 10^-6 ^and distance between SNPs <10 cM.

**Epistasis**	**SNP_1**	**SNP_2**	**-log_10 _*p*-value**	**r^2^**	**Distance**
**AxA**	3512	3652	6.59	1.6 × 10^-2^	14 cM
	3083	5023	6.32	5.8 × 10^-5^	Diff. chr
	1002	5847	6.21	1.6 × 10^-4^	Diff. chr
**AxD**	1546	1652	6.58	2 × 10^-2^	19.1 cM
	1843	3248	6.02	1.3 × 10^-3^	Diff. chr
**DxA**	1308	1411	6.12	1.1 × 10^-2^	10.3 cM
**DxD**	3939	5524	6.32	7.2 × 10^-4^	Diff. chr

r^2 ^is the correlation between SNPs

### Comparison with the true QTLs

Comparisons with the true simulated loci showed a similar overall performance of Blossoc and SMA, and major differences related to the trait modeling (Table 3). Trait modeling reduced false positives but also power, mainly for loci with small effect. Blossoc had greater power than SMA to detect loci with an effect size between 0.5 and 1%. Threshold relaxation increases Blossoc power without incorporating any additional false positives. Using a higher threshold on Blossoc with raw data, similar power is achieved for medium to large effect loci. Nevertheless, this power is reduced for small effect loci, but allowing for a better control of false positives. Bagging performs similarly to approaches explicitly using the pedigree.

**Table 3:** Proportion of true positives and power to detect true QTLs in each analysis.

			**Power^2^**			
			
**Data**	**Approach**	**TP^1^**	**overall**	**>1%**	**[0.5–1]%**	**<0.5%**
**Raw**	**Blossoc (15)^3^**	0.49	0.74	1	1	0.70
	**Blossoc (65)**	0.68	0.42	0.87	0.71	0.24
	**SMA**	0.61	0.65	1	0.71	0.55
	**SMA Bagging**	0.87	0.40	1	0.71	0.18
**Corrected**	**Blossoc (15)**	1	0.33	0.87	0.57	0.15
	**Blossoc (12)**	1	0.40	1	0.71	0.18
	**SMA**	1	0.35	0.87	0.43	0.21

1 – The proportion of true positives is the proportion of detected QTLs that were true. Positive match between detected and true QTLs was based on a maximum distance of 5 cM.

2 – Overall power is the proportion of true loci that were detected. This power is further subdivided according to QTL categories based on the true effect size: >1% (8 QTLs), between 0.5 and 1% inclusive (7 QTLs), and <0.5% (33 QTLs) of the total phenotypic variance.

3 – Numbers between brackets for Blossoc are the threshold used for selecting peaks.

## Conclusion

Both SMA and haplotype-based methods performed similarly in detecting the most strongly associated SNPs. The use of raw data increased the number of positive results in comparison to corrected phenotypes. The concordance among matching SNPs increased when using adjusted data compared to raw data. The largest discrepancies found in this study were between different phenotype modeling rather than between single and multiple marker approaches. Thus, model choice should be carefully considered in this kind of studies. Bagging seems a promising approach to control for inflation and false positives when the population structure is hidden.

## List of abbreviations used

SNP: single-nucleotide polymorphism; QTL: quantitative trait loci; SMA: single marker association; GWAS: genome-wide association studies; LD: linkage disequilibrium; HQ: Hannan and Quinn criteria.

## Competing interests

The authors declare that they have no competing interests.

## Authors' contributions

MCL and NN carried out research. MPE supervised research. All wrote the manuscript.

## Supplementary Material

Additional file 1 **SNPs associated to the phenotype by SMA and Blossoc methods using raw data**. Threshold for SMA is P < 10^-8 ^and for Blossoc is HQ Score ≥ 15. 1 – Bootstrap posterior probabilities of 1000 models for SMA raw of the 33 SNPs that pass the threshold of -log_10_(p) ≥ 8. * – Significant associations, considering a BPP > 0.25 for SMA raw, decreased the number of associated SNPs to 15. Considering an adjusted threshold for Blossoc raw ≥ 65, to account for the inflation caused by the population structure, the number of significant associated SNPs decreased to 19. Click here for file

Additional file 2 **Total CPU time required for the analyses, including the significance tests**. 1 – a)1^st ^step: 600 bins (179,700 interactions * 4 tests); b) 2^nd ^step: top interactions with -log_10_(p) >3 for 675 pairwise locations to refine (67,500 interactions: 4 tests with an average of 169 pairwise locations to refine within 1 cM). 2 – Correction with mixed model including sex, generation and infinitesimal effects using QxPak. Note: Model aggregation using 1000 bootstrap samples on the 33 putative QTLs of the SMA raw took 4 h 37 m 46 s (Additional file 1). Analyses were performed on a Linux server with dual Xeon processors and 8 Gb RAM. From the programs used, only Blossoc was specifically dedicated to GWAS. The R-Scripts for SMA, bagging and epistasis were not optimized to speed-up calculation. Qxpak is a program initially dedicated to QTL mapping in livestock. SMA on raw data outperformed the two other approaches in the initial genome-scan. Blossoc is very fast considering a haplotype-based method. QxPak ran in a reasonable time considering its internal correction for the population structure. However, this time precludes using computationally intensive procedures to control false positives. The bagging took about 4 h 38 m (33 candidate SNPs, 1000 models, on average 13.4 SNPs per model). The proper calibration of the BPP will require S more times (S = number of simulations done for calibration). Nevertheless, strong code optimization will be straightforward and will dramatically reduce computing time. The two-step strategy for epistasis was reasonably efficient given the large number of interaction terms that were tested. Click here for file

Additional file 3 **Methods for Interactions within and between loci and with sex**. Analyses of the interaction within locus (dominance), between loci (epistasis) and with sex were performed using only the corrected SMA procedure implemented on an R script. We used residual data from a mixed model including sex, generation and pedigree. This approximation is necessary given the computational burden of between loci interactions. For dominance and sex, we tested the effects of dominance or of the interaction between additive and sex against a model incorporating only an additive effect. No procedure for controlling false positives was done and only best results with strong -log_10_(p) are reported here. For the epistasis, a two step strategy based on LD between SNPs was used. First, we subdivided the genome in 1 cM bins, and tested epistasis between the bins. In each bin, the SNP with the highest average LD (r^2^) with all the other SNPs in the bin was chosen as the representative SNP of the bin (tagSNP) and used for the interaction testing. Cockerham parameterization of the model was used [11] and all four possible interactions (axa, axd, dxa, dxd) were tested against a reduced model incorporating only the additive and dominance effect. Then, for each type of interaction, the ones with a -log_10_(p) ≥ 3 were selected in order to refine the location of the interacting loci. We refined these locations by evaluating interactions between all SNPs contained in the two bins initially detected to have some significant interactions. Further, we ranked the results for each type of interactions according to their significance value. Nonetheless, we control potential LD effect by removing SNPs in a minimal distance of 10 cM from the top ranked list (-log_10_(p) ≥ 6). Click here for file
