# Supplementary material for: Data modeling as a main source of discrepancies in single and multiple marker association methods
Source: BMC Proc. 2009 Feb 23;3(Suppl 1):S9. doi: 10.1186/1753-6561-3-s1-s9 (PMC2654503; doi:10.1186/1753-6561-3-s1-s9)
Supplement: Additional file 1 — SNPs associated to the phenotype by SMA and Blossoc methods using raw data. Threshold for SMA is P < 10-8 and for Blossoc is HQ Score ≥ 15. 1 – Bootstrap posterior probabilities of 1000 models for SMA raw of the 33 SNPs that pass the threshold of -log10(p) ≥ 8. * – Significant associations, considering a BPP > 0.25 for SMA raw, decreased the number of associated SNPs to 15. Considering an adjusted threshold for Blossoc raw ≥ 65, to account for the inflation caused by the population structure, the number of significant associated SNPs decreased to 19. [file 1753-6561-3-S1-S9-S1.pdf]

Chr	Blossoc raw		SMA raw		
	SNP	Score	SNP	$-\log_{10} p$-value	BPP[1]
**1**	46	46.55	52	9.89	0.04
	104	50.20			
	200	215.66*	196	52.24*	1
	265	131.37*			
			323	16.87	0.1
	416	98.12*	415	23.51*	0.67
	476	72.78*			
	599	97.76*	599	25.70	0
	684	34.66			
	778	51.70	778	14.89*	1
	986	38.66	1019	8.97	0
**2**	1268	94.58*	1271	15.84*	1
	1483	113.25*	1483	28.31*	1
	1704	97.42*	1673	15.37*	0.74
			1758	14.98*	0.79
	1884	31.47			
	1974	24.24			
**3**	2134	65.79*	2149	17.25*	0.89
	2211	48.48	2211	13.93	0.01
	2272	43.96			
	2409	38.62	2410	11.04	0.05
	2598	37.87			
	2702	37.37			
	2754	71.15*	2772	11.37	0.03
			2860	8.39	0
**4**	3032	237.36*	3048	35.91*	1
			3103	15.44	0
	3267	90.74*	3255	14.61	0.04
	3356	86.58*	3341	18.57*	0.66
	3509	126.93*	3507	21.17	0.19
	3638	94.36*	3649	20.59	0.08
	3765	182.98*	3765	44.90*	1
	3881	99.32*	3891	15.63	0.02
	3952	103.19*	3953	17.00*	0.99
**5**	4163	42.12	4164	8.99*	0.33
	4278	26.01			
	4355	46.17			
	4413	32.22			
	4482	28.38			
	4600	34.14			
	4696	36.54	4705	10.14	0.07
	4779	33.14	4775	10.27	0.08
	4940	94.76*	4935	23.70*	1
**6**	5073	30.09	5073	8.68	0.11
	5178	47.05	5149	10.84*	0.32
			5206	9.18	0.17
	5295	37.43			
	5354	20.08			

	5428	30.52			
	5530	37.07	5489	8.61	0.04
	5593	25.55			
	5693	21.24			
	5813	29.30			
	5973	15.62			
