# Supplementary material for: Data modeling as a main source of discrepancies in single and multiple marker association methods
Source: BMC Proc. 2009 Feb 23;3(Suppl 1):S9. doi: 10.1186/1753-6561-3-s1-s9 (PMC2654503; doi:10.1186/1753-6561-3-s1-s9)
Supplement: Additional file 2 — Total CPU time required for the analyses, including the significance tests. 1 – a)1st step: 600 bins (179,700 interactions * 4 tests); b) 2nd step: top interactions with -log10(p) >3 for 675 pairwise locations to refine (67,500 interactions: 4 tests with an average of 169 pairwise locations to refine within 1 cM). 2 – Correction with mixed model including sex, generation and infinitesimal effects using QxPak. Note: Model aggregation using 1000 bootstrap samples on the 33 putative QTLs of the SMA raw took 4 h 37 m 46 s (Additional file 1). Analyses were performed on a Linux server with dual Xeon processors and 8 Gb RAM. From the programs used, only Blossoc was specifically dedicated to GWAS. The R-Scripts for SMA, bagging and epistasis were not optimized to speed-up calculation. Qxpak is a program initially dedicated to QTL mapping in livestock. SMA on raw data outperformed the two other approaches in the initial genome-scan. Blossoc is very fast considering a haplotype-based method. QxPak ran in a reasonable time considering its internal correction for the population structure. However, this time precludes using computationally intensive procedures to control false positives. The bagging took about 4 h 38 m (33 candidate SNPs, 1000 models, on average 13.4 SNPs per model). The proper calibration of the BPP will require S more times (S = number of simulations done for calibration). Nevertheless, strong code optimization will be straightforward and will dramatically reduce computing time. The two-step strategy for epistasis was reasonably efficient given the large number of interaction terms that were tested. [file 1753-6561-3-S1-S9-S2.doc]

|  | **SMA raw** | **SMA corrected (QxPak)** | **Blossoc**  **raw / corrected** | **Epistasis with^1^ SMA (R script)** |
| --- | --- | --- | --- | --- |
| **Analysis** | 3m 57s | 2h 13m 10s | 13m 48s | 1. 8h 8m 3s 2. 3h 6m 47s |
| **Correction with mixed model ^2^** | **-** | Included in the analysis | **- /** 16s | 16s |
| **Total time** | **3m 57s** | **2h 13m 10s** | **13m 48s / 14m 04s** | **11h 15m 06s** |
