# Supplementary material for: Data modeling as a main source of discrepancies in single and multiple marker association methods
Source: BMC Proc. 2009 Feb 23;3(Suppl 1):S9. doi: 10.1186/1753-6561-3-s1-s9 (PMC2654503; doi:10.1186/1753-6561-3-s1-s9)
Supplement: Additional file 3 — Methods for Interactions within and between loci and with sex. Analyses of the interaction within locus (dominance), between loci (epistasis) and with sex were performed using only the corrected SMA procedure implemented on an R script. We used residual data from a mixed model including sex, generation and pedigree. This approximation is necessary given the computational burden of between loci interactions. For dominance and sex, we tested the effects of dominance or of the interaction between additive and sex against a model incorporating only an additive effect. No procedure for controlling false positives was done and only best results with strong -log10(p) are reported here. For the epistasis, a two step strategy based on LD between SNPs was used. First, we subdivided the genome in 1 cM bins, and tested epistasis between the bins. In each bin, the SNP with the highest average LD (r2) with all the other SNPs in the bin was chosen as the representative SNP of the bin (tagSNP) and used for the interaction testing. Cockerham parameterization of the model was used [11] and all four possible interactions (axa, axd, dxa, dxd) were tested against a reduced model incorporating only the additive and dominance effect. Then, for each type of interaction, the ones with a -log10(p) ≥ 3 were selected in order to refine the location of the interacting loci. We refined these locations by evaluating interactions between all SNPs contained in the two bins initially detected to have some significant interactions. Further, we ranked the results for each type of interactions according to their significance value. Nonetheless, we control potential LD effect by removing SNPs in a minimal distance of 10 cM from the top ranked list (-log10(p) ≥ 6). [file 1753-6561-3-S1-S9-S3.doc]

Additional file 3

Analyses of the interaction within locus (dominance), between loci (epistasis) and with sex were performed using only the corrected SMA procedure implemented on an R script. We used residual data from a mixed model including sex, generation and pedigree. This approximation is necessary given the computational burden of between loci interactions. For dominance and sex, we tested the effects of dominance or of the interaction between additive and sex against a model incorporating only an additive effect. No procedure for controlling false positives was done and only best results with strong -log_10_(p) are reported here. For the epistasis, a two step strategy based on LD between SNPs was used. First, we subdivided the genome in 1cM bins, and tested epistasis between the bins. In each bin, the SNP with the highest average LD (r^2^) with all the other SNPs in the bin was chosen as the representative SNP of the bin (tagSNP) and used for the interaction testing. Cockerham parameterization of the model was used [11] and all four possible interactions (axa, axd, dxa, dxd) were tested against a reduced model incorporating only the additive and dominance effect. Then, for each type of interaction, the ones with a -log_10_(p) ≥ 3 were selected in order to refine the location of the interacting loci. We refined these locations by evaluating interactions between all SNPs contained in the two bins initially detected to have some significant interactions. Further, we ranked the results for each type of interactions according to their significance value. Nonetheless, we control potential LD effect by removing SNPs in a minimal distance of 10 cM from the top ranked list (-log_10_(p) ≥ 6).
